# Expansion of low-price cigarette market and its implications for cigarette tax revenue: Evidence from Bangladesh

**DOI:** 10.18332/tid/209145

**Published:** 2025-10-18

**Authors:** Md. Nazmul Hossain, Rumana Huque, S. M. Abdullah, Nigar Nargis

**Affiliations:** 1 Department of Economics, University of Dhaka, Dhaka, Bangladesh; 2 ARK Foundation, Dhaka, Bangladesh; 3 American Cancer Society, Atlanta, United States

**Keywords:** cigarette tax revenue, low priced cigarettes, Bangladesh, tax simulation, tobacco industry response

## Abstract

**INTRODUCTION:**

In Bangladesh, a significantly lower minimum retail price and preferential ad valorem tax rate for low-price cigarettes incentivized manufacturers to avoid tax by expanding the low-price cigarettes market. The effect of this industry response on government tax revenue has not been quantified yet. This study aims to fill this gap.

**METHODS:**

Using cigarette sales data (2019–2020) of British American Tobacco (BAT) Bangladesh in the WHO Tobacco Tax Simulation Model, we estimated the gap of actual from potential revenue by simulating four counterfactual scenarios involving government tax interventions and cigarette manufacturers’ decision to expand low-price cigarette sales. We analyzed optimal government policy response vis-à-vis manufacturers’ actions in a game theoretic framework based on a payoff matrix of tax revenue and industry revenue.

**RESULTS:**

The revenue gap due to expansion of low-price cigarette sales (scenario 1) was BDT 22.1 billion (US$ 0.26 billion; US$ 1≈ BDT85 in Year 2020), equivalent to around 10% of the collected revenue in 2019–2020. Due to lower minimum price of low-price cigarettes (scenario 2), the revenue gap was BDT 14.7 billion (US$ 0.17 billion). The revenue gap was BDT 30.5 billion (US$ 0.36 billion) for the lower minimum price and lower excise tax rate of low-price cigarettes (scenario 3). The revenue gap due to lower minimum price, lower excise tax rate of low-price cigarettes and low-price cigarette sales expansion (scenario 4) was BDT 49.4 billion (US$ 0.58 billion).

**CONCLUSIONS:**

In Bangladesh, revising the tiered excise tax structure by raising prices in the low-tier and unifying tax rates across tiers can curb tax avoidance, boost government revenue, and promote public health.

## INTRODUCTION

The use of tobacco products, in smoke or smokeless form, is a major risk factor for many chronic non-communicable diseases and one of the major causes of premature death. Bangladesh, a low-to-middle-income country with a very high-density population, bears a huge burden of the tobacco epidemic. Around 35.3% of all adults (aged ≥15 years) in Bangladesh use tobacco products (smoked or smokeless), whereas the prevalence of cigarette smoking among adults is 14.0% (28.7% among men and 0.2% among women)^1^. The high prevalence of tobacco use has significant health and economic costs for Bangladesh. In 2018, the use of tobacco caused the premature deaths of nearly 126000 people in Bangladesh, accounting for 13.5% of deaths from any cause^2^. Also, more than 61000 children (<15 years) suffered from various diseases resulting from exposure to secondhand smoke. The net economic loss due to tobacco-related deaths and diseases in Bangladesh was calculated to be around BDT 76.5 billion (about US$ 0.7 billion)^2^.

Increases in cigarette taxes can reduce smoking while generating larger tax revenue^3^.Tobacco taxes are a major source of revenue for the Bangladesh government. In 2017–2018, the National Board of Revenue (NBR), Bangladesh, collected around 11% of its total tax revenue from tobacco. A large portion, around 96.1%, of tobacco tax revenue comes from cigarettes^4^. Raising cigarette prices through increased taxation could lead to a win-win-win situation in Bangladesh – it would reduce cigarette consumption, increase tax revenue, and potentially decrease socioeconomic inequities, given the higher price sensitivity of cigarette smoking among people in lower socio-economic status who also tend to smoke at higher rates^5,6^. But the complex tiered ad valorem cigarette tax structure in Bangladesh, with a low minimum retail price for each tier, has reduced the effectiveness of tobacco tax in reducing cigarette smoking and created an opportunity for its manufacturers to avoid taxes.

Bangladesh’s cigarette excise tax structure is one of the most complex tax systems in the world. Cigarettes are taxed at differential rates in four price tiers – low, medium, high, and premium – depending on the price range of each tier. The excise tax for each tier, known as supplementary duty (SD), is imposed as a percentage of final retail price specified by the NBR, which acts as the tax base. Moreover, a value-added tax (VAT) of 15% and a health development surcharge (HDS) of 1% are imposed on all manufactured cigarettes’ retail price (Supplementary file Table S1). Cigarette prices in Bangladesh are also among the lowest in the world (Supplementary file Table S1). In 2018 and 2020, the prices of both premium and cheapest brands of cigarettes in Bangladesh were significantly lower than the global averages^7,8^.

A tiered tax structure that imposes lower excise taxes on low-priced cigarettes incentivizes manufacturers to prioritize low-priced sales, leading to tax avoidance that results in revenue losses for the government. Over the years, the excise tax for the lowest tier has remained lower than that for the three higher tiers, leading to the expansion of the market for low-priced cigarettes. Furthermore, the price difference between low- and medium-tier cigarettes grew in 2019–2020 (Supplementary file Table S1), providing an opportunity for cigarette companies to launch new low-price brands and expand the low-tier cigarette market. To our knowledge, no systematic analysis has been done about the impact of this lowprice cigarette market expansion on government’s tax revenue in Bangladesh.

We aimed to estimate the change in cigarette tax revenue in Bangladesh due to the increase in low-tier cigarette sales by British American Tobacco (BAT), the leading cigarette company in Bangladesh. We also shed light on how government cigarette pricing and tax policies can help minimize tax avoidance by cigarette companies that tend to expand the low-price cigarette market with lower tax liability. This evidence can assist the government in making informed decisions about tobacco taxation to avert revenue loss not only in Bangladesh but also in other settings with a tiered tax system.

## METHODS

### Data and measures

We used data on the tier-specific prices and sales volumes of BAT for 2018–2019 and 2019–2020 with tier-wise tax rates (SD, VAT and HDS). All data were sourced from the NBR, Bangladesh.

We focused exclusively on BAT’s data in our analysis because it has emerged as the dominant player in Bangladesh’s cigarette market. While the low-price segment was historically led by few manufacturers, BAT significantly expanded its presence in recent years. Between 2009 and 2016, BAT’s annual profit rose by 121%, largely due to a 103% increase in sales volume^9^. BAT’s market share in sales volume also grew from 49% in 2006–2007 to 78% in 2019–2020, and it accounted for around 82% of NBR’s total cigarette tax revenue in 2019–20 (Supplementary file Table S2). Given this market dominance, BAT data provide a reliable basis for assessing industry-wide impacts.

An important measure to evaluate the effect of cigarette price or tax changes on consumption and tax revenue is the price elasticity of demand. A larger absolute value of the price elasticity of demand results in a greater impact on consumption and government revenues, *ceteris paribus*. In this study, we used the recent estimate of price elasticity of cigarette demand in the low and medium tiers in Bangladesh, which is -0.168^10^.

The market for low-price cigarettes has significantly expanded, as indicated by BAT’s sales volume share rising from 36.8% in 2018–2019 to 69.0% in 2019– 2020 (Table 1). Concurrently, the share of mid-tier brands drastically fell from 37.3% to 10.1%. The lower price and lower tax rate on low-tier cigarette brands created a reduced tax burden for these brands, which prompted cigarette manufacturers like BAT to increase their sales volume of low-tier cigarette brands. This situation is expected to result in a revenue loss for the government due to the significant differences in price levels and SD rates between the low and medium tiers.

**Table 1:** Sales volume share of British American Tobacco (BAT) in Bangladesh, by tier in 2018–2019 and 2019–2020

*Tiers and brands*	*Sales volume share (%)*	
	*2018–2019*	*2019–2020*
Premium	15.2	10.1
High	10.7	10.8
Medium	37.3	10.1
Low	36.8	69.0

Source: Authors’ calculations from National Board of Revenue data.

### Analytical framework

We applied the World Health Organization (WHO) tax simulation model (TaXSiM) to simulate the effects of changes in prices, tax rates, and tax structure on cigarette sales, government tax revenue, and industry revenue across price tiers. Details of the TaXSiM simulation calculations are provided in Supplementary file Material^11,12^. The analysis relies on the following assumptions:

- Smokers’ brand or price-tier preferences are positively correlated with their income levels, with higher income individuals tending to prefer higher priced brands.
- Each price tier has a distinct price elasticity of demand, reflecting variation in consumer responsiveness to price changes across tiers. The price elasticity of low-and-medium tier cigarettes is assumed to be -0.168^10^.
- Smokers who switch to lower priced brands primarily substitute within adjacent tiers – that is, they are more likely to trade down to the next lowest price category rather than making a substantial shift across tiers. Given the study’s focus on expansion of low-price, we assumed that this primarily resulted from smokers switch from medium tier.
- High/premium-tier prices, tax rates and sales across scenarios are unchanged.
- Producer’s prices remain unchanged following change in tax policy.
- Tax burden is fully passed on to the cigarette consumers through price increase.
- Overall inflation does not affect the cigarette consumption decision, because of high prevalence of single-stick sales and yearly adjustments of price by the government.

### Counterfactual scenarios for simulation

Using the TaXSiM model, we first calculated the baseline (status quo) tax and industry revenue. Then, we assessed potential tax and industry revenue across four distinct counterfactual scenarios. In all these scenarios, the prices, tax rates and sales of premium and high tier are assumed to remain the same as in the baseline. The description and objective of each scenario is given in Table 2. Finally, we measured the revenue gap for each scenario using the difference between potential revenues from the simulated scenarios and the baseline revenues.

**Table 2:** Description and objective of different scenarios in the simulation model

*Scenarios*	*Description of scenarios*	
	*Government policy* ^a^	*Industry action* ^b^
Baseline (status quo)	Low price (BDT 37) for low-tier cigarettes Lower excise tax rates (55%) for low-tier cigarettes	Low-tier expansion
Scenario 1	Same as baseline	No low-tier expansion
Scenario 2	Price increased	Low-tier expansion
Scenario 3	Price increased Tax increased	Low-tier expansion
Scenario 4	Price increased Tax increased	No low-tier expansion

a Price increased: implies price of low tier cigarettes increased from BDT 37 to BDT 45; Tax increased: implies excise tax rates increased from 55% to 65% for low-tier cigarettes.

b Low tier expansion: implies decrease in share of medium tier cigarettes sales and increase in share of low tier cigarettes sales. BDT: 1000 Bangladeshi Takas about US$8.2.

*Scenario 1*

Under this scenario we assumed that the mid-tier market share of BAT had not been lowered and the low-tier market share had not been increased by large amount in 2019–2020 compared to 2018–2019, and that the total sales in 2019–2020 remained the same. Had the market share of the medium tier not been lowered in 2019–2020, more mid-tier cigarette sales would have been realized. The number of cigarettes consumed under this scenario may, however, vary depending on smokers’ response to the price differential between low and mid-tier cigarettes. The formula used to estimate the medium-tier cigarette sales is as follows:


$$S_{m}^{*}=(\text{S}\times \text{r})\left(1+\frac{P_{m}^{R}-P_{l}^{R}}{P_{l}^{R}}\cdot \text{ε}\right)$$ (1)

where $P_{i}^{R}$ is the actual retailer price of tier i for all *i=l,m* where *l* is low-tier and *m* is medium-tier. Also, *r* is the market share of medium tier in 2018–2019 and *S* is the total sales in 2019–2020, $S_{m}^{*}$ is the estimated sales of medium-tier cigarettes in 2019–2020 and *ε* is the price elasticity of low-and-medium tier cigarette demand which is -0.168. In this scenario, since we are assuming that the proportion of medium tier sales in 2019–2020 is the same as it was in 2018–2019, we calculated *(S*×*r)*. This amount is then adjusted for the price differentials between low and medium tier cigarettes using the elasticity value. That is why *(S*×*r)* is multiplied by the second part in the above equation. We also assumed that there was no change in tax structure, i.e. the price range of each tier, excise tax, VAT, and HDS rates remain the same as in the baseline.

*Scenario 2*

Under scenario 2, we assumed that low-tier cigarette price was BDT 45 instead of BDT 37, which would have made the price differential between the medium and low tier BDT 18 instead of BDT 26. The assumed increase in price will have a negative effect on the sales of low-tier cigarettes and the new sales of low-tier cigarettes was calculated as follows:


$$S_{l}^{*}=S_{l}\left(1+\frac{P_{l}^{R*}-P_{l}^{R}}{P_{l}^{R}}\cdot \text{ε}\right)$$ (2)

where $S_{l}^{*}$ is the estimated sales, *S_l_* is the actual sales, $P_{l}^{R}$ is the actual retail price and $P_{l}^{\mathrm{R*}}$ is the assumed retail price of low-tier cigarettes. Similar to scenario 1, *ε* is the price elasticity of cigarette demand which is -0.168.

*Scenario 3*

Under scenario 3, along with an increased price of BDT 45 for low-tier cigarettes instead of BDT 37, it is assumed that the government has imposed a uniform ad valorem excise tax (65%) for all tiers. Since the price change remained the same as in scenario 2, the same formula for $S_{l}^{*}$ is used to estimate the sales of low-tier cigarettes.

*Scenario 4*

This is the combination of policy intervention of scenario 1 and scenario 3. Under scenario 4, it is assumed that BAT had not expanded the sales of low-tier cigarette sales (as in scenario 1) and that the government had imposed a uniform ad valorem excise tax (65%) for all tiers along with a price increase for low-tier cigarettes (as in scenario 3).

### Game theoretical analysis

We analyzed the estimations under the counterfactual scenarios using a strategic decision-making model framework to find the best strategy for the government to increase tax revenue^13^. We designed the analysis as matrix payoff game with two players. Each player will try to maximize their payoffs considering the strategies of other players. For example, consider the following simple setup with two players A and B, both having two strategies (1 and 2). Table 3 contains the payoff matrix where the first element in each cell is the payoff of A and the second element is the payoff of B.

**Table 3:** Payoff matrix of two players with two strategies each

		*B*	
		*1*	*2*
**A**	**1**	*a_11_, b_11_*	*a_12_, b_21_*
	**2**	*a_21_, b_12_*	*a_22_, b_22_*

Considering player B’s strategy 1, A will be better off choosing strategy 1 if a_11_>a_21_, but A will be better off choosing strategy 2 if a_11_<a_21_. Again, considering player B’s strategy 2, A will be better off choosing strategy 1 if a_12_>a_22_, but A will be better of choosing strategy 2 if a_12_<a_22_. Similarly, we can analyse the best strategy for play B, considering player A’s strategy. Dominant strategy is the strategy that provides better payoffs than any other strategy a player might choose, no matter what strategy the other player chooses. Here, if a_11_>a_21_ and a_12_>a_22_, then no matter what strategy B chooses, player A’s dominant strategy will be 1. To find the likely outcome in a game-theoretic setup, game theorists use a concept of ‘Nash equilibrium’. A Nash equilibrium is a state in a game where no player can improve their payoff by unilaterally changing their strategy, given that all other players keep their strategies unchanged.

In practice, the two players are the government and BAT. Each player has two strategies. First player, BAT, can expand sales of low-price cigarettes or it can refrain from doing so. Second player, the government, can increase low-tier cigarette prices along with imposing a uniform ad valorem excise tax of 65% or they can keep the low-tier cigarette price low along with a lower ad valorem excise tax for low-tier cigarettes. The industry revenues and tax revenues from the above simulated scenarios can be considered as the payoffs for players, respectively, for the BAT and the government.

## RESULTS

### Baseline results

**Figure 1:**
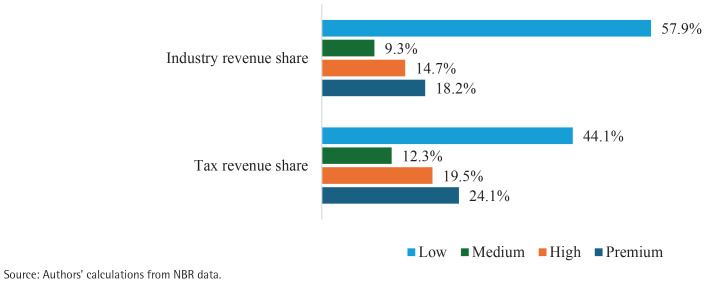
Share of cigarette tax revenue and industry revenue, by tier, from BAT’s cigarette brands

In the status quo, overall government tax revenue was around BDT 219.9 billion and the industry revenue was around BDT 68.4 billion. Even though the prices and excise taxes of low-tier cigarettes were lower in 2019–2020 than those of other tiers, the lion’s share of tax revenue (57.9%) and industry revenue (44.1%) came from the low-tier cigarettes sales (Figure 1). This is due to the large share of low-tier cigarettes in total sales of cigarettes (69%).

### Comparison of simulation results of each scenario with baseline results

As shown in Table 4, the estimated tax revenue under scenario 1 is BDT 241.9 billion which indicates a revenue gap of BDT 22.1 billion from the baseline tax revenue. This implies that government tax revenue would have been around BDT 22.1 billion higher if BAT had not expanded its low-tier sales. Also, BAT’s expansion of low-tier cigarette sales helped the company to earn more, as the simulation shows that industry revenue increased by BDT 1.7 billion. Therefore, the increase in low-tier cigarettes sales benefited BAT by reducing their tax liability and increasing their revenue while the government had lost tax revenue.

**Table 4:** Estimated government tax revenue (billion BDT) and industry revenue for BAT under all scenarios

*Scenarios*	*Tax revenue*	*Tax revenue gap*	*Industry revenue (BAT)*	*Industry revenue gap*
Baseline	219.9	-	68.4	-
Scenario 1	241.9	22.1	66.8	-1.7
Scenario 2	234.6	14.7	74.4	6.0
Scenario 3	250.3	30.5	58.7	-9.7
Scenario 4	269.3	49.4	63.2	-5.3

Source: Authors’ calculations from NBR data using TaXSiM model. US$ 1 ≈ BDT85 in Year 2020.

Under scenario 2, the government revenue gap is estimated to be around BDT 14.7 billion. This suggests that had the government increased the price of low-tier cigarettes, tax revenue would have increased by BDT 14.7 billion. Interestingly, BAT’s industry revenue is also estimated to increase by BDT 6.0 billion, which might induce BAT to expand its low-tier market share more. This finding is very important because it shows that only increasing price without increasing the tax rate might not be an effective policy intervention to control low-tier market expansion and tax avoidance.

Under scenario 3, the estimated government revenue gap is BDT 30.5 billion and BAT’s industry revenue is BDT 9.7 billion lower than the baseline. Hence, the government policy intervention under scenario 3, which is increasing the low-tier cigarettes price and imposing a uniform excise tax, would have resulted in less incentive for BAT to expand its market share of low-tier cigarettes.

The outcome pattern under scenario 4 is similar to that under scenario 3. The estimated government revenue gap is BDT 49.4 billion. Also, BAT’s revenue is estimated to be reduced by BDT 5.3 billion. Hence, again, the findings show that a price increase along with an excise tax increase would have resulted in less incentive for BAT to expand the low-tier market.

### Game theoretical analysis of the results

Table 5 represents the game and the simulated results together. In the payoff matrix, each cell (baseline, scenario 1, scenario 3, and scenario 4) has two payoffs. The first amount in each cell, which is the industry revenue for that specific scenario, represents the payoffs for BAT. Similarly, the second amount in each cell, which is the tax revenue for that specific scenario, represents the payoff for the government.

**Table 5:** Game-theoretical representation (in payoff matrix) of results under different scenarios

*BAT*	*Government*	
	*Substantially low price along with lower ad valorem* *excise tax for low tier (status quo policy)*	*Increase low-tier price along with uniform ad* *valorem excise tax (policy intervention 2)*
Expansion of low tier (status quo strategy)	Baseline 68.4; 219.9	Scenario 3 58.7; 250.3
Not expanding the low tier	Scenario 1 66.8; 241.9	Scenario 4 63.2; 269.3

From the government’s perspective, if BAT expands the low tier, the government would be better off by choosing policy intervention 2 since its payoff (tax revenue) for policy intervention 2 (BDT 250.3 billion) is more than the payoff for the status quo policy (BDT 219.9 billion). A similar conclusion is true for the government when BAT does not expand the low tier. Therefore, policy intervention 2 is the ‘dominant strategy’ for the government.

Under the government’s current tax policy, BAT has an incentive to introduce a new brand in the low tier since its payoff (industry revenue) in the baseline (BDT 68.4 billion) is greater than the payoff when it does not introduce a new brand in the low tier (BDT 66.8 billion). But under policy intervention 2, BAT’s payoff when it does not expand the low tier (BDT 63.2 billion) is greater than the payoff when it expands the low tier (BDT 58.7 billion). Therefore, under policy intervention 2, BAT would be better off by not expanding the market share of the low tier.

Since policy intervention 2 is the dominant strategy for the government, the government should enact this policy. In this situation, BAT would be better off when it does not expand the market share of the low tier. Therefore, the outcomes of scenario 4, where the government imposes a uniform ad valorem excise tax along with a higher price for low-tier cigarettes and BAT does not expand the low tier, are the equilibrium outcomes for both. This is also the Nash equilibrium of the game.

## DISCUSSION

Over time, the retail prices of each tier of cigarettes in Bangladesh remained significantly low compared to other countries. Manufacturers were able to alter their production decisions, like introducing a new brand in the low tier, to avoid higher tax liabilities, causing revenue loss to the government^4^. Adding to these existing challenges, in 2019–2020, the Bangladesh government increased the price differential between the low and medium tiers, making low-tier cigarettes relatively cheaper. The policy changes in 2019–2020 as mentioned above induced BAT, a major player in the tobacco industry of Bangladesh, to expand the market share of low-tier cigarettes and lower the market share of medium-tier cigarettes. Within one fiscal year from 2018–2019 to 2019–2020, there was nearly a two-fold increase in the share of low-tier cigarettes in BAT’s total cigarette sales with concomitant decline in the share of medium-tier cigarettes by more than two-thirds.

We estimate that due to the reduction in the share of medium-tier cigarettes and the expansion of the share of low-tier cigarettes by BAT, the Bangladesh government missed a large revenue potential of BDT 22.1 billion. Had the government increased the minimum retail price of low-tier cigarettes in 2019–2020 without any change in excise tax from the status quo as a policy measure in anticipation of the expansion of low-tier cigarette market, there would have been a gain in tax revenue by BDT 14.7 billion. However, this policy measure would have also generated larger industry revenue for BAT by BDT 6.0 billion, creating an incentive to BAT to further expand low-tier cigarette sales, which would have widened the tax revenue gap further. Our analysis suggests that to achieve the government’s desired result of dissuading expansion of the low-tier cigarette market in addition to earning higher tax revenue, a price increase in low-tier cigarettes must be accompanied by an increase in the excise tax rate in the low tier. When an increase in the minimum retail price of low-tier cigarettes is combined with a uniform 65% ad valorem excise tax rate for all tiers implying an increase in the tax rate in the low tier from 55% to 65%, the potential revenue gain becomes much larger to the amount of BDT 30.5 billion when BAT continues to expand the low-tier cigarette market and BDT 49.4 billion when the share of low-tier cigarettes remains unchanged. BAT’s revenues under both scenarios become lower than the baseline results which are expected to demotivate BAT to expand the low-tier market share.

The simulation results from Bangladesh highlighted the notable fiscal consequences of tobacco taxation structures. Previous research indicates that, in response to tax increases, the tobacco industry introduces new brands, price segments, and products to counteract the beneficial effects of tobacco control^14,15^. In Thailand and Spain, the industry has launched low-priced products to avoid taxation^16,17^. A study from Bangladesh revealed that, in light of growing price differences among brands, the tobacco industry provided low-priced cigarettes to boost sales by encouraging smokers to switch from bidis (locally made, inexpensive cigarettes)^9^. This strategic behavior stems from the fact that in low- and middle-income countries, the industry focuses on a volume expansion model, maintaining low prices to increase sales^18^.

In the context of Bangladesh, findings from the game theoretical analysis of this study confirms that imposing a uniform ad valorem excise tax along with an increase in the minimum retail price of low-tier cigarettes will be a dominant strategy for the government, leading the cigarette manufacturer to refrain from expanding the low-tier market while also ensuring a significant increase in tax revenue. This strategy would reduce incentives for expansion into the low-tier segment by manufacturers, and secure substantial improvements in both tax revenues and public health outcomes. Historically, the industry has demonstrated significant adaptability, introducing new products, price segments, and marketing strategies to undermine the intended public health impact of tobacco taxation. Without a well-designed, forward-looking tax structure, the industry can exploit loopholes to maintain or even expand its consumer base, particularly among price-sensitive populations. These insights suggest that to ensure a successful tobacco control, government must anticipate and pre-empt strategic industry responses and design tax structures accordingly to strengthen tobacco control efforts. This includes adopting uniform tax structures, narrowing price gaps between brands, and strengthening regulatory oversight to reduce tax evasion.

Implementing a uniform tax structure would benefit public health and simplify administration for Bangladesh’s tax authorities by reducing enforcement complexity and opportunities for tax evasion. Raising the minimum retail price and narrowing price gaps can further deter smoking initiation and encourage quitting, particularly among youth and low-income populations. However, these reforms may encounter strong opposition from industry stakeholders. With sufficient political commitment, stakeholder participation, and public support, the proposed changes are both manageable administratively and consistent with the country’s long-term health and revenue objectives.

### Strengths and limitations

This is one of the few studies, perhaps the only one from Bangladesh, that quantifies the tobacco industry’s response to government tax interventions. The strength of this study lies in its use of a widely accepted simulation method and a suitable framework for analyzing strategic interactions. Nonetheless, it does have some limitations. One major limitation of the analysis in this study is based on the cigarette prices and sales data for BAT and may not be generalizable to other cigarette manufacturers in the country. Further analysis can be done using data from other manufacturers. Also, the revenue gap is estimated for one fiscal year only. To estimate the cumulative government tax revenue gap, further research can be undertaken with updated data from subsequent fiscal years. In addition, the analysis does not account for potential residual confounding factors such as changes in consumer behavior unrelated to tax policy, income effect, the influence of illicit trade, or substitution toward other tobacco products. These factors may affect the accuracy of the estimated impact and warrant further investigation.

## CONCLUSIONS

The preferential treatment of low-price cigarettes in a tiered excise tax structure in Bangladesh encourages cigarette manufacturers to expand the market for lowprice and more affordable cigarettes to the detriment of public health. Increasing price in the low-price tier in combination with unifying the differential cigarette excise tax rates across four cigarette price tiers can help mitigate cigarette manufacturer’s incentive for tax avoidance and increase government’s revenue while reducing cigarette consumption and improving public health.

## Supplementary Material



## Data Availability

The data supporting this research cannot be made available for privacy or other reasons. The cigarette sales data obtained from the National Board of Revenue (NBR), Bangladesh, are restricted. Anyone wishing to access the data would need to reach out to NBR directly to obtain permission to use data.
