# Supplementary material for: Expansion of low-price cigarette market and its implications for cigarette tax revenue: Evidence from Bangladesh
Source: Tob Induc Dis. 2025 Oct 18;23:10.18332/tid/209145. doi: 10.18332/tid/209145 (PMC12535224; doi:10.18332/tid/209145)
Supplement: Supplementary file 1 [file TID-23-159-s1.pdf]

Supplementary table 1. Cigarette prices and tax rates, by tier, in Bangladesh from 2006-07 to 2021-22

Year	Premium		High		Medium		Low	
	**Price**	**SD**	**Price**	**SD**	**Price**	**SD**	**Price**	**SD**
2006-07	≥ 30.00	57%	18.00-24.99	55%	10.50-12.49	52%	5.25-6.24	32%
2007-08	≥ 35.00	57%	19.00-26.49	55%	12.50-13.49	52%	6.00-6.99	32%
2008-09	≥ 41.00	57%	21.00-28.00	55%	13.25-14.25	52%	6.50-7.50	32%
2009-10	≥ 46.25	57%	23.25-29.25	55%	16.25-17.25	52%	7.25-8.75	32%
2010-11	≥ 52.00	58%	27.00-32.00	56%	18.40-19.00	53%	8.40-9.15	33%
2011-12	≥ 60.00	60%	32.36-36.00	58%	22.50-23.00	55%	11.00-11.30	36%
2012-13	≥ 66.00	61%	35.20-39.50	59%	24.75-25.25	56%	12.10-12.30	39%
2013-14	≥ 80.00	61%	42.00-45.00	59%	28.00-30.00	56%	13.69-13.91	39%
2014-15	≥ 90.00	61%	50.00-54.00	61%	32.50-35.00	60%	15.00-16.50	43%
2015-16	≥ 70.00	64%	≥ 45.00	62%	≥ 45.00	62%	18	48%
2016-17	≥ 70.00	65%	≥ 45.00	63%	≥ 45.00	63%	23	51%
2017-18	≥ 70.00	65%	≥ 45.00	63%	≥ 45.00	63%	27	53%
2018-19	101	65%	75	65%	48	65%	32	55%
2019-20	123	65%	93	65%	63	65%	37	55%
2020-21	128	65%	97	65%	63	65%	39	57%
2021-22	135	65%	102	65%	63	65%	39	57%

Note: Prices are in Bangladeshi taka. In addition to Supplementary Duty (SD), Value Added Tax (VAT) of 15 percent is applied to all tiers. Moreover, from 2014-15, Health Development Surcharge (HDS) of 1 percent is applied to all tiers.
Source: Nargis et al (2019) and National Board of Revenue, Bangladesh.

Supplementary figure 1. Distribution of 20-cigarette pack prices from 144 countries in 2018 and 2020

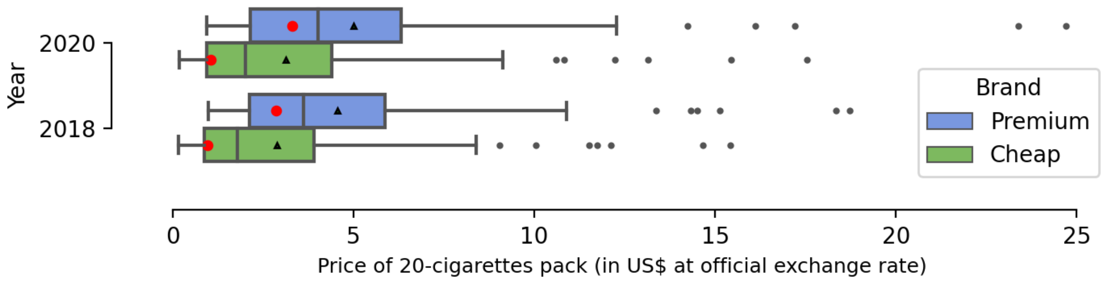

Note: The red dots show prices in Bangladesh, and black triangles are the mean prices for 144 countries for the respective categories. In the years 2018 and 2020, the prices in Bangladesh (red dots) for both premium and cheap brands of cigarettes are significantly lower than the average (black triangles) and the median (vertical line inside the boxes) cigarette prices from 144 countries.

Source: Authors' calculations from *WHO Report on the Global Tobacco Epidemic*, 2019 and 2021

Supplementary table 2. Tier-wise Cigarette Market Share and Contribution to Tax Revenue of British American Tobacco Bangladesh

Year	Tier-wise Market Share				Total Market Share	BAT's Share in Total Tax Revenue
	Premium	High	Medium	Low		
2006-07	100%	92%	54%	0%	49%	69%
2007-08	99%	87%	61%	3%	44%	67%
2008-09	99%	93%	60%	1%	40%	65%
2009-10	99%	97%	61%	7%	41%	68%
2010-11	99%	97%	52%	9%	35%	62%
2011-12	98%	98%	45%	15%	36%	60%
2012-13	98%	99%	58%	26%	47%	68%
2013-14	98%	99%	58%	30%	47%	67%
2014-15	97%	99%	59%	38%	50%	68%
2015-16	97%	98%	64%	48%	55%	68%
2016-17	96%	98%	66%	58%	63%	72%
2017-18	97%	99%	68%	62%	68%	75%
2018-19	98%	100%	59%	59%	66%	72%
2019-20	97%	100%	65%	75%	78%	82%

Source: National Board of Revenue, Ministry of Finance, Government of Bangladesh, 2021

Supplementary Material: Detailed TaXSiM simulation calculations

Tax type, tax base, tax tier, and total tax

Currently in Bangladesh the NBR levies three types of taxes on cigarette sales: the excise tax or supplementary duty (SD), the value added tax (VAT), and the health development surcharge (HDS). For all types of taxes, the base is the market retail price of a ten-stick cigarette pack (P_R).

In Bangladesh the excise tax is ad valorem in nature. Assume the ad valorem excise tax for a ten-stick pack is denoted as E_b, whose rate is defined as t_e. Since there are four tiers (premium, high, medium, and low) in Bangladesh, there are four excise tax rates (e_k) where $k = 1, 2, 3, 4$. The tier of each brand is defined using the information of the retailer ` and the price range of each tier. Thus, the amount of ad valorem excise tax for a ten-stick cigarette pack is calculated as follows:

$$E_b = e_k * P_b^R$$

The rate of excise tax differs for different tiers. But the VAT rate (v) is uniform for all tiers. The amount of VAT per unit (V) is calculated as follows:

$$V_b = v * P_b^R$$

Like the VAT rate, the HDS rate (h) is also flat across all tiers, and the amount of health development surcharge (H) for a ten-stick pack is calculated as follows:

$$H_b = h * P_b^R$$

Thus, the total tax on a ten-stick pack of a cigarette brand is determined as follows:

$$T_b = E_b + V_b + H_b$$

The excise tax revenue, the VAT revenue, and the HDS revenue for each brand (b) can also be calculated as follows:

$$E_b^{Total} = E_b * S_b$$

$$V_b^{Total} = V_b * S_b$$

$$H_b^{Total} = H_b * S_b$$

where S_b is the sales volume of a ten-stick pack of a brand (b). Subsequently, the total amount of tax revenue from a cigarette brand is calculated as follows:

$$TR_b = T_b * S_b \equiv E_b^{Total} + V_b^{Total} + H_b^{Total}$$

Consumer and producer price per ten-pack of cigarettes and distribution margin

In this simulation model, there are three components in the final retail price of a cigarette brand that a consumer pays. They are as follows:

$$P_b^R = P_b^P + M_b + T_b$$

where P_b^P is the producer price, M_b is the distribution margin, and T_b is the total tax per pack of cigarette brands b. From the factory gate to the point of retail sale, cigarettes pass through different market actors (including wholesalers, distributors, and retailers) in the supply chain, and each actor receives a margin from the cigarette price. For simplicity, rather than separating the margin of each actor in the supply chain, this study combines them into the total "distribution margin (M_b)". The distribution margin information is not available from the individual actors. So, for simplicity, the distribution margin rate is assumed to be $t_M = 10\%$. Thus, the distribution margin for a cigarette brand is calculated as follows:

$$M_b = t_M * P_b^R$$

This simulation framework requires the producer price to calculate the producer's revenue. Based on the final retail price, tax, and distribution margin, the producer price per pack of cigarettes can be calculated as follows:

$$P_b^P = P_b^R - M_b - T_b$$

Aggregation by tier

Assume that there are n_k number of brands in tier k. Since the sales value and sales volume data are known for each brand, and the tier of each brand has been defined, the average retail price for each tier can be calculated by dividing the total sales value of all brands in a tier by the total sales volume of each tier as follows:

$$P_k^R = \frac{\sum_{b=1}^{n_k}(P_b^R * S_b)}{\sum_{b=1}^{n_k} S_b}$$

Using the average price for each tier, the excise tax, VAT, HDS, and total tax by tier for a 10-stick pack can be calculated as follows:

$$E_k = e_k * P_k^R$$

$$V_k = v * P_k^R$$

$$H_k = h * P_k^R$$

$$T_k = E_k + V_k + H_k$$

Now, the excise tax revenue, the VAT revenue, and the HDS revenue for each tier (k) can be calculated as follows:

$$E_k^{Total} = E_k * S_k \equiv \sum_{b=1}^{n_k} (E_b * S_b)$$

$$V_k^{Total} = V_k * S_k \equiv \sum_{b=1}^{n_k} (V_b * S_b)$$

$$H_k^{Total} = H_k * S_k \equiv \sum_{b=1}^{n_k} (H_b * S_b)$$

where $S_k = \sum_{b=1}^{n_k} S_b$ is the total sales volume of each tier.

The total tax revenue—including excise tax, VAT, and HDS—for each tier can be calculated as follows:

$$TR_k = E_k^{Total} + V_k^{Total} + H_k^{Total} \equiv \sum_{b=1}^{n_k} TR_b$$

where $k = 1, 2, 3, 4$ denotes the four tiers of cigarettes and n_k denotes the number of cigarette brands in tier k. Finally, total excise tax revenue (E), total VAT revenue (V), total HDS revenue (H), and total tax revenue (TR) from cigarettes are calculated as follows:

$$E = \sum_{k=1}^{4} E_k^{Total} \equiv \sum_{b=1}^{N} E_b^{Total}$$

$$V = \sum_{k=1}^{4} V_k^{Total} \equiv \sum_{b=1}^{N} V_b^{Total}$$

$$H = \sum_{k=1}^{4} H_k^{Total} \equiv \sum_{b=1}^{N} H_b^{Total}$$

$$TR = \sum_{k=1}^{4} TR_k \equiv \sum_{b=1}^{N} TR_b$$

where $N = \sum_{k=1}^{4} n_k$.

Now, to calculate the industry revenue, the producer revenue and distribution margin must be calculated. For each tier, the distribution margin and the producer price for a ten-stick pack will be:

$$M_k = t_M * P_k^R$$

$$P_k^P = P_k^R - M_k - T_k$$

Hence, the total distribution margin (M_k^{Total}), total producer revenue (C_k^{Total}), and total industry revenue (IR_k) for each tier will be as follows:

$$M_k^{Total} = M_k * S_k$$

$$C_k^{Total} = P_k^P * S_k$$

$$IR_k = M_k^{Total} + C_k^{Total}$$

Finally, the total industry revenue (IR) will be:

$$IR = \sum_{k=1}^{4} IR_k$$

© 2025 Hossain M.N. et al.
